# [^18^F]FDG PET/CT versus [^18^F]FDG PET/MRI in the diagnosis of lymph node metastasis in nasopharyngeal carcinoma: a systematic review and meta-analysis

**DOI:** 10.3389/fmed.2024.1450526

**Published:** 2024-10-16

**Authors:** Junfang Lei, Xu Li, Wenbo Xue, Xinrui Qian, Tong Wang, Yunuo Xiang, Yangchun Zhang, Meixing Chen, Zhaohui Liu

**Affiliations:** Department of Otorhinolaryngology, Affiliated Hospital of Zunyi Medical University, Zunyi, China

**Keywords:** fluorodeoxyglucose, positron emission tomography, lymph node metastasis, nasopharyngeal carcinoma, meta-analysis

## Abstract

**Purpose:**

This meta-analysis aimed to evaluate the comparative diagnostic accuracy of [^18^F]FDG PET/CT versus [^18^F]FDG PET/MRI in identifying lymph node metastases in individuals with nasopharyngeal carcinoma.

**Methods:**

A comprehensive search was executed across PubMed, Embase, and Web of Science through September 2023 to identify studies evaluating the diagnostic precision of [^18^F]FDG PET/CT and [^18^F]FDG PET/MRI in detecting lymph node metastasis in nasopharyngeal carcinoma. Sensitivity and specificity were assessed through the DerSimonian-Laird method, incorporating the Freeman-Tukey transformation.

**Results:**

The meta-analysis encompassed nine articles, involving a total of 916 patients. The overall sensitivity and specificity of [^18^F]FDG PET were 0.95 (95%CI: 0.88–1.00) and 0.95 (95%CI: 0.84–1.00). The overall sensitivity of [^18^F]FDG PET/CT was 0.94 (95%CI, 0.85–0.99), whereas [^18^F]FDG PET/MRI achieved a sensitivity of 1.00 (95%CI, 0.94–1.00). The findings reveal that [^18^F]FDG PET/CT demonstrates comparable sensitivity to [^18^F]FDG PET/MRI (*p* = 0.20). The overall specificity of [^18^F]FDG PET/CT was 0.94 (95%CI, 0.82–1.00), whereas [^18^F]FDG PET/MRI exhibited a specificity of 0.98 (95%CI, 0.93–1.00). Additionally, the results suggest that [^18^F]FDG PET/CT offers similar specificity to [^18^F]FDG PET/MRI (*p* = 0.11).

**Conclusion:**

[^18^F]FDG PET demonstrates high sensitivity and specificity in identifying lymph node metastasis in nasopharyngeal carcinoma. Furthermore, [^18^F]FDG PET/CT exhibits comparable sensitivity and specificity to [^18^F]FDG PET/MRI.

**Systematic review registration:**

https://www.crd.york.ac.uk/prospero/display_record.php?RecordID=496006, PROSPERO (CRD42024496006).

## 1 Introduction

Nasopharyngeal carcinoma (NPC), predominantly found in Southeast Asia and Southern China, is known for its asymptomatic nature and high incidence of early lymph node involvement (1, 2). Lymph node metastasis in NPC represents a critical prognostic factor, influencing treatment and outcomes (3–5). Therefore, early diagnosis is extremely important for the treatment and prognosis of NPC. Thus, early detection of lymph node metastasis is crucial for effective management and improved survival rates (5).

Conventional diagnostic methods, including computed tomography (CT), magnetic resonance imaging (MRI), and biopsies, are widely used but exhibit certain limitations. CT scans, although quick and accessible, often struggle with specificity in early-stage disease (6). MRI, while providing superior soft-tissue contrast, cannot reliably distinguish between benign and malignant lesions, a critical aspect where PET imaging shows greater accuracy (7). Biopsies, while definitive, are invasive and carry risks of complications and sampling errors (8).

The advent of [^18^F]fluorodeoxyglucose (FDG) positron emission tomography combined with CT ([^18^F]FDG PET/CT) and PET/MRI has revolutionized the diagnostic landscape in NPC (9, 10). These modalities amalgamate metabolic and anatomical imaging, enhancing the detection and characterization of lymph node metastases. [^18^F]FDG PET/CT, combining PET’s metabolic imaging with CT’s anatomic resolution, offers significant advantages in staging and detecting metastases (11). On the other hand, PET/MRI, merging PET’s metabolic insights with MRI’s superior soft-tissue contrast, potentially provides even greater diagnostic precision (12, 13). However, a debate persists regarding the superiority of PET/CT over PET/MRI in this context, with each modality having its proponents and specific clinical scenarios (14, 15).

Therefore, we aimed to perform a meta-analysis to evaluate the diagnostic performance of [^18^F]FDG PET and to compare the effectiveness of [^18^F]FDG PET/CT and PET/MRI in diagnosing lymph node metastasis in NPC.

## 2 Methods

This meta-analysis was conducted in accordance with the Preferred Reporting Items for Systematic Reviews and Meta-Analyses of Diagnostic Test Accuracy (PRISMA-DTA) guidelines (16). The protocol for this meta-analysis is registered with PROSPERO (CRD42024496006).

### 2.1 Search strategy

A comprehensive literature search was performed across PubMed, Embase, and Web of Science, encompassing publications available up to September 2023. This search utilized key terms including “Positron-Emission Tomography,” “Lymph Node Metastasis,” and “Nasopharyngeal Carcinoma.” For further detail strategy, refer to Supplementary Table S1. Additionally, the reference lists of the selected studies were meticulously reviewed to identify any suitable articles.

### 2.2 Inclusion and exclusion criteria

Inclusion criteria for this meta-analysis was applied as follows: Population (P): patients with nasopharyngeal carcinoma; Intervention (I): [^18^F]FDG PET/CT or [^18^F]FDG PET/MRI; Comparator (C): in studies comparing both modalities, PET/CT and PET/MRI were directly compared, while non-comparative studies were also included; Outcome (O): diagnostic accuracy in detecting lymph node metastasis; Study design (S): prospective or retrospective studies.

Exclusions were applied to duplicate articles, abstract-only publications, editorial comments, letters, case reports, reviews, meta-analyses, and irrelevant titles or abstracts. Studies lacking complete or clear data necessary for calculating the sensitivity or specificity of the evaluated imaging techniques were also excluded. In cases of potential patient population overlap, only the most recent publication was included.

### 2.3 Quality assessment

A Quality Assessment of Diagnostic Accuracy Studies-2 (QUADAS-2) tool was used by two researchers to evaluate the quality of the included studies (17). A QUADAS-2 framework consists of four critical domains: patient selection, index test, reference standard, and flow and timing. The risk of bias was categorized into high, low, and unclear risk.

### 2.4 Data extraction

Data was gathered independently from chosen articles by two researchers. The data included author, year of publication, imaging test type, study characteristics (country, study design, analysis, and reference standard), characteristics of patients (number of patients, clinical indication, mean/median age, and previous treatment), and technical aspects (scanner modality, image analysis, and radiotracer dose).

In cases of disagreement, the researchers engaged in discussion to reach a consensus, thereby ensuring the accuracy of the extracted data.

### 2.5 Statistical analysis

The DerSimonian and Laird method was used to assess specificity and sensitivity, and then transformed through the Freeman-Tukey double arcsine transformation. Based on the Cochrane Q test and I^2^ statistics, heterogeneity within and across groups was evaluated. In cases where significant heterogeneity was detected (*p* > 0.10 or I^2^ > 50%), meta-regression and sensitivity analyses were conducted.

Publication bias was evaluated using both a funnel plot and Egger’s test. *p* < 0.05 was deemed statistically significant for all statistical tests. The statistical analyses were conducted using R software version 4.2.3, which is designed for statistical computing and graphics.

## 3 Results

### 3.1 Study selection

**Figure 1:**
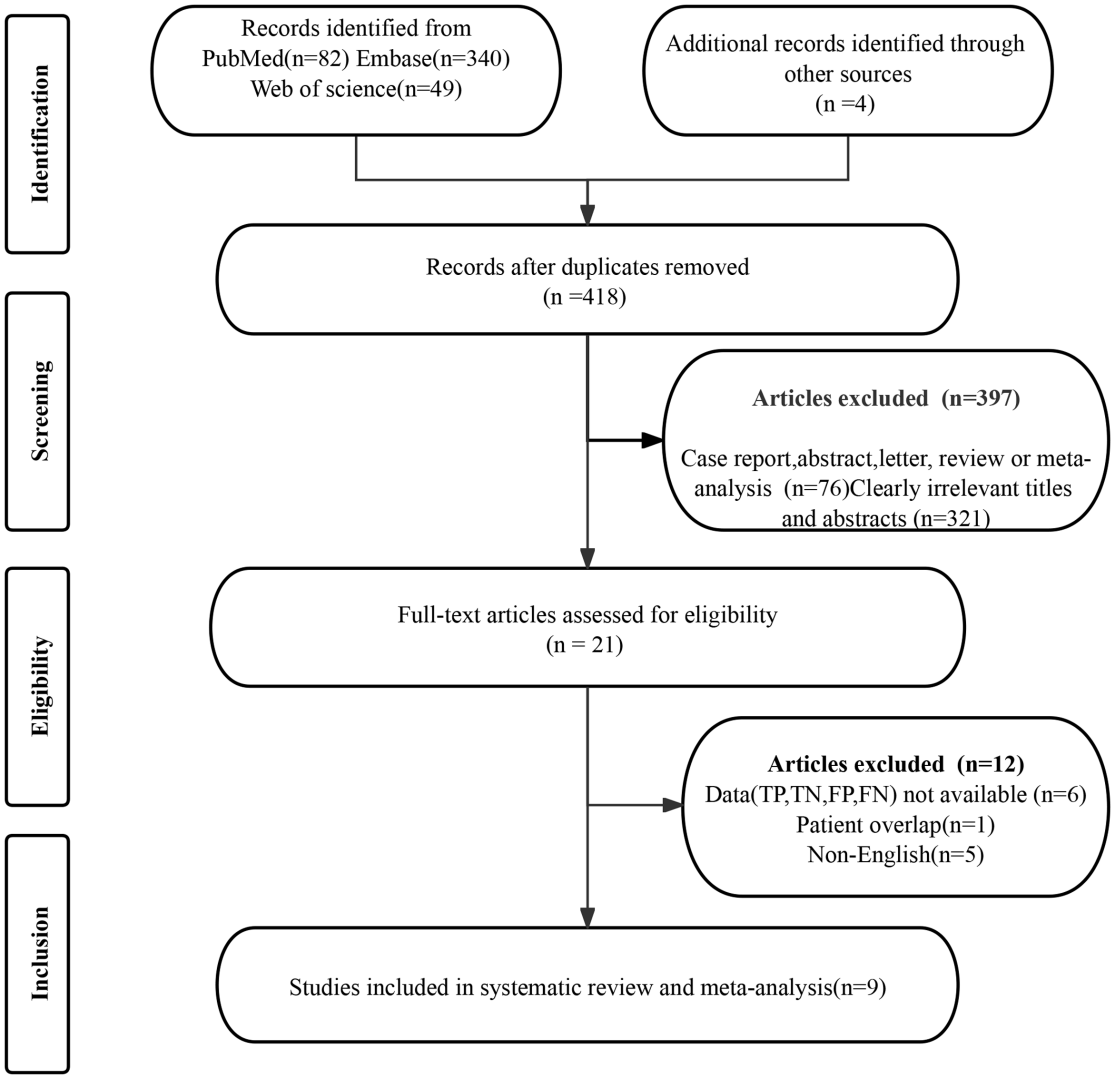
PRISMA flow diagram illustrating the study selection process.

The initial search yielded 418 publications, of which 397were excluded for not meeting the eligibility criteria. Further detailed evaluation of the remaining 21articles led to the exclusion of 12 studies, due to unavailable data (TP, FP, FN, and TN) (*n* = 6), patient overlap (*n* = 1), and non-English (*n* = 5). Consequently, 9 articles evaluating the diagnostic performance of [^18^F]FDG PET/CT and/or [^18^F]FDG PET/MRI were included in the meta-analysis (18–26). The article selection process is illustrated in Figure 1, following the PRISMA flow diagram format.

### 3.2 Study description and quality assessment

The nine selected studies encompassed a total of 916 nasopharyngeal cancer patients, with individual study sizes ranging from 27 to 218. Among these, four studies were retrospective, while five were prospective. Regarding analysis methods, six studies conducted patient-based analysis and three opted for lesion-based analysis. As for the reference standard, two studies utilized pathology, six combined pathology and/or follow-up imaging, and one relied only on follow-up imaging. The characteristics of the studies and the techniques used in [^18^F]FDG PET/CT and [^18^F]FDG PET/MRI are detailed in Tables 1, 2.

**Table 1:** Study and patient characteristics of the included studies for [^18^F]FDG PET/CT and [^18^F]FDG PET/MRI.

Author	Year	Type of imaging test	Study characteristics				Patient characteristics			
			Country	Study design	Analysis	Reference standard	No. of patients	Clinical indication	Mean/Median age	Previous treatment
Chen et al.	2006	PET/CT	Taiwan	Retro	PB	Follow-up imaging	70	Initial stage or Post-treatment stage	Median ± SD: (46.3 ± 15.6)	NA
Comoretto et al.	2008	PET/CT	Italy	Retro	PB	Pathology or follow-up imaging	63	Post-treatment stage	Mean (range): 52 (13–79)	Chemotherapy and radiation therapy
Ng et al.	2009	PET/CT	Taiwan	Pro	PB	Pathology or follow-up imaging	150	Initial stage	Mean (range): 48.17 (17–84)	NO
Moon et al.	2016	PET/CT	South Korea	Retro	LB	Pathology and follow-up imaging	41	Post-treatment stage	Mean = 50.0 ± 13.2 (range, 12–78)	Radiotherapy or chemoradiotherapy
Chan et al.	2018	PET/CT and PET/MRI	Taiwan	Pro	PB	Pathology and follow-up imaging	113	Post-treatment stage	Median ± SD: (51 ± 12)	Radiation therapy or chemotherapy or chemoradiotherapy
Xiao et al.	2021	PET/CT	China	Pro	LB	Pathology or follow-up imaging	218	Initial stage	Mean (range): 44.5 (35–51)	Intensity-modulated radiation therapy and/or radiotherapy and/or chemoradiotherapy
Yang et al.	2022	PET/CT	China	Pro	PB	Pathology	174	Post-treatment stage	Median (range): 48 (13–69)	Intensity modulated radiotherapy and chemotherapy
Piao et al.	2022	PET/MRI	China	Retro	PB	Pathology or follow-up imaging	60	Initial stage or Post-treatment stage	Mean (range): 51 (26–73)	NA
Ding et al.	2022	PET/CT	China	Pro	LB	Pathology	27	Initial stage	Mean ± SD: (53 ± 10)	NO

PB, patient-based; LB, lesion-based; Pro, prospective; Retro, retrospective; NA, not available.

**Table 2:** Technical aspects of included studies.

Author	Year	Types of imaging tests	Scanner modality	Radiotracer dose	Image analysis	TP	FP	FN	TN
Chen et al.	2006	PET/CT	Discovery LS, GE Medical Systems, Waukesha, WI, United States	370 MBq	Visual	19	0	0	1
Comoretto et al.	2008	PET/CT	Discovery LS; GE Healthcare, Milwaukee, Wis	350–400 MBq	Visual	20	1	1	41
Ng et al.	2009	PET/CT	Discovery ST 16, GE Healthcare, Milwaukee, United States	370 MBq	Visual	5	1	2	142
Moon et al.	2016	PET/CT	GE STE scanner (GE Healthcare, Milwaukee, WI; 35 scans) or GE Discovery LS scanner (GE Healthcare, Milwaukee, WI; 16 scans)	370 MBq	Semiquantitative	21	3	8	42
Chan et al.	2018	PET/CT and PET/MRI	Biograph mCTscanner (Siemens Medical Solutions, Malvern, PA, United States) and Biograph mMR (Siemens Healthcare, Erlangen, Germany)	370 MBq	Visual	4	1	0	108
Xiao et al.	2021	PET/CT	Discovery ST 16; GE Healthcare	5.55 MBq/kg	Visual	143	51	25	217
Yang et al.	2022	PET/CT	Discovery ST-16 (GE Medical Systems, Milwaukee)	3.7 Mbq/kg	Visual	129	18	3	74
Piao et al.	2022	PET/MRI	GE Health-care	NA	Visual	18	2	0	40
Ding et al.	2022	PET/CT	uMI780, United Imaging Healthcare	1.85 MBq/kg	Semiquantitative	228	20	6	31

TP, true positive; TN, true negative; FP, false positive; FN, false negative; NA, not available.

**Figure 2:**
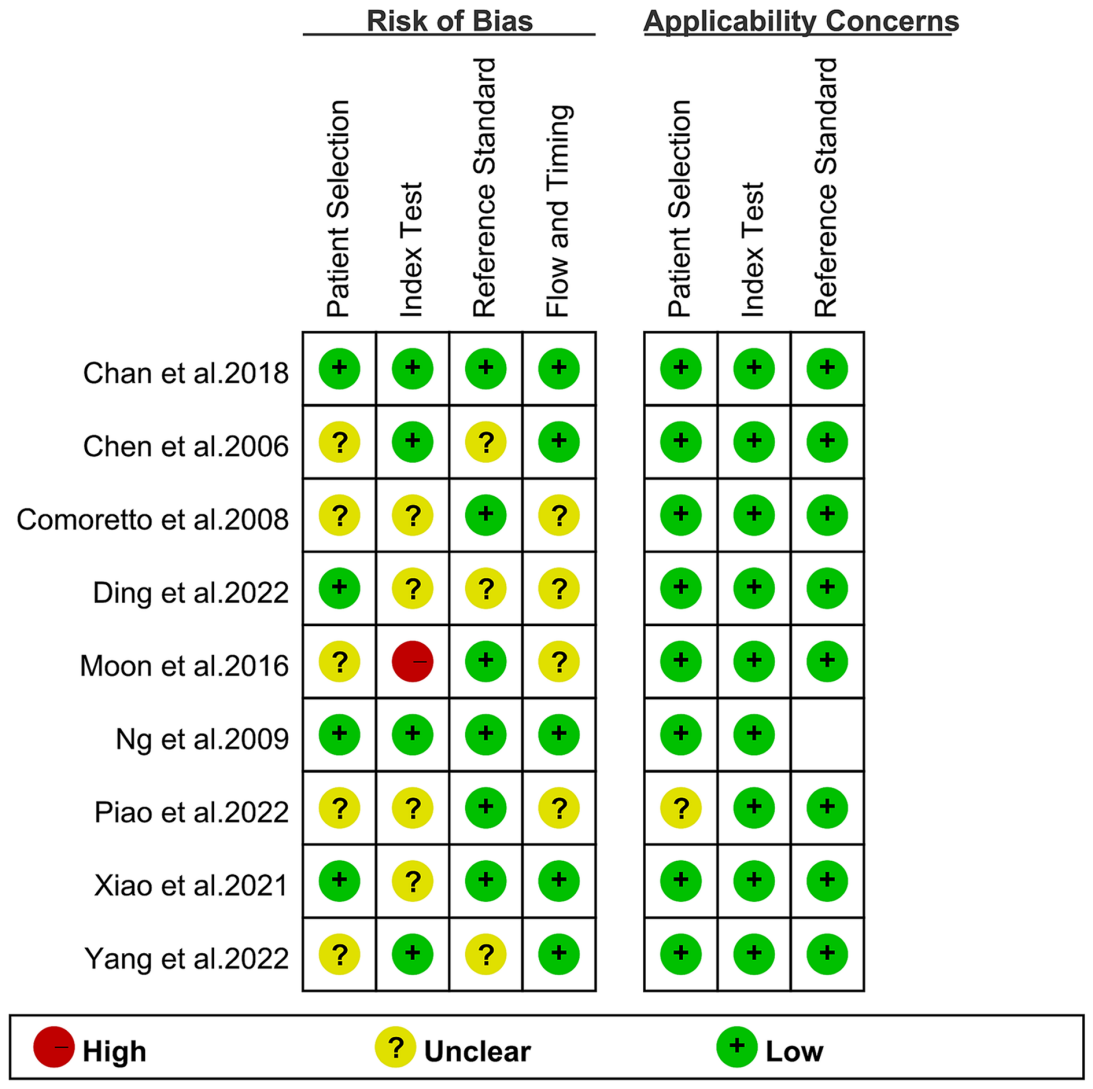
Risk of bias and applicability concerns of the included studies using the Quality Assessment of Diagnostic Accuracy Studies-2 (QUADAS-2) tool.

The risk of bias in each study, as assessed by the QUADAS-2 tool, is depicted in Figure 2. Regarding the index test, one study was classified as “high risk” due to the absence of pre-determined cut-off values. Overall, the quality assessment revealed no significant concerns regarding the quality of the included studies.

### 3.3 The diagnostic performance of [^18^F]FDG PET in detecting lymph node metastasis in nasopharyngeal carcinoma

**Figure 3:**
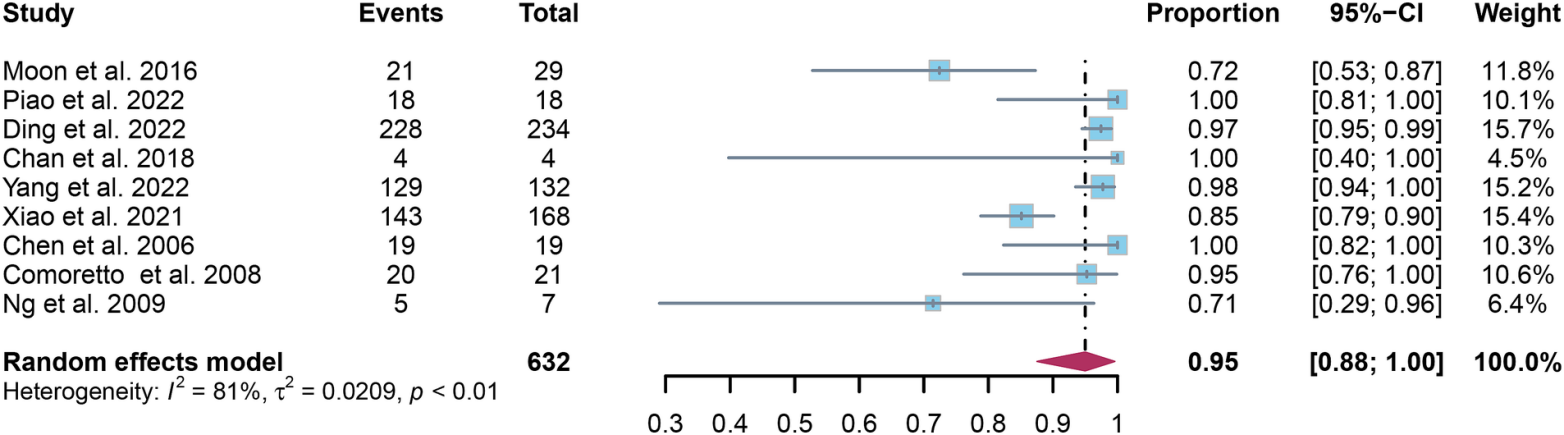
Forest plot of [^18^F]FDG PET sensitivity in detecting lymph node metastasis in nasopharyngeal carcinoma.

**Figure 4:**
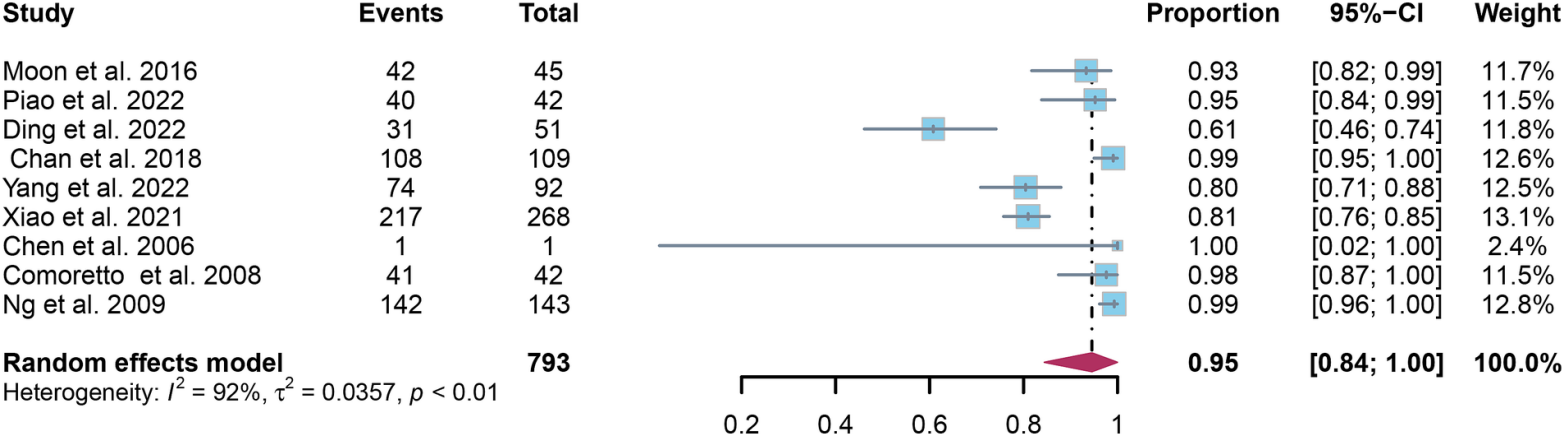
Forest plot of [^18^F]FDG PET specificity in detecting lymph node metastasis in nasopharyngeal carcinoma.

A total of nine studies were incorporated into the analysis. The pooled sensitivity and specificity of [^18^F]FDG PET for detecting lymph node metastasis in nasopharyngeal carcinoma were 0.95 (95%CI: 0.88–1.00) and 0.95 (95%CI: 0.84–1.00), respectively, as illustrated in Figures 3, 4.

**Figure 5:**
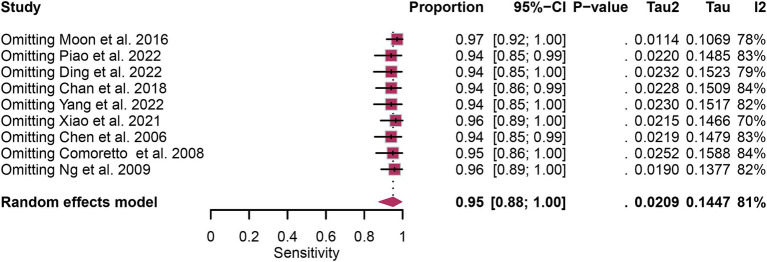
Sensitivity analysis of [^18^F] FDG PET for lymph node metastasis sensitivity in nasopharyngeal carcinoma.

**Figure 6:**
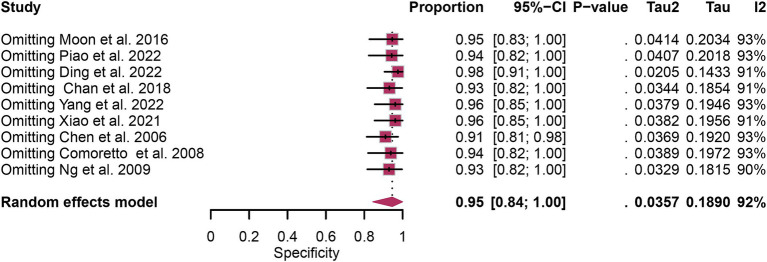
Sensitivity analysis of [^18^F] FDG PET for lymph node metastasis specificity in nasopharyngeal carcinoma.

In the pooled analysis of [^18^F]FDG PET’s overall sensitivity and specificity, the I^2^ values were 81 and 92%, respectively. Meta-regression and sensitivity analysis for sensitivity identified no potential sources of heterogeneity. However, for specificity, meta-regression indicated that the analytical method (*p* = 0.03) might contribute to heterogeneity. Sensitivity analysis did not reveal any potential sources of heterogeneity (Table 3). The outcomes from the sensitivity analysis were stable, demonstrating minimal variation, with sensitivity ranging from 0.94 to 0.97 and specificity from 0.91 to 0.98 (Figures 5, 6).

**Table 3:** Subgroup analysis and meta-regression analysis.

Covariate	Studies, *n*	Sensitivity (95%CI)	*p*-value	Specificity (95%CI)	*p*-value
Number of patients included			0.52		0.65
>100	4	0.93 (0.79–1.00)		0.93 (0.79–1.00)	
≤100	5	0.96 (0.85–1.00)		0.95 (0.76–1.00)	
Region			0.92		0.51
Europe	1	0.95 (0.78–1.00)		0.98 (0.87–1.00)	
Non-Europe	8	0.95 (0.86–1.00)		0.94 (0.82–1.00)	
Study design			0.96		0.46
Retrospective	4	0.95 (0.80–1.00)		1.00 (0.97–1.00)	
Prospective	5	0.95 (0.86–1.00)		0.88 (0.71–0.98)	
Analytical method			0.24		0.03
Patient-based	6	1.00 (0.97–1.00)		1.00 (0.93–1.00)	
Lesion-based	3	0.88 (0.70–0.99)		0.80 (0.60–0.94)	
Image analysis			0.57		0.19
Semiquantitative	2	0.89 (0.55–1.00)		0.79 (0.41–1.00)	
Visual	7	0.97 (0.89–1.00)		0.98 (0.90–1.00)	

### 3.4 Comparing the sensitivity of [^18^F]FDG PET/CT and [^18^F]FDG PET/MRI for detecting lymph node metastasis in nasopharyngeal carcinoma

**Figure 7:**
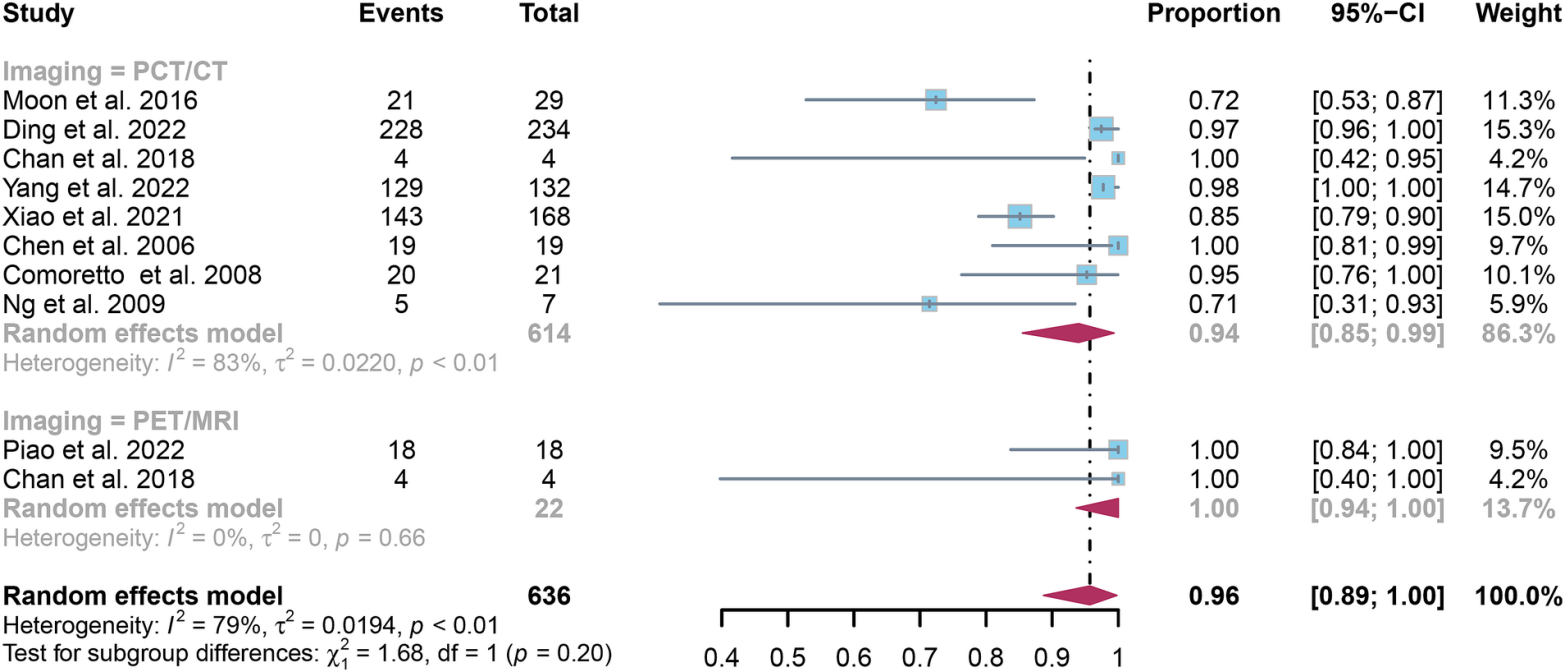
Forest plot comparing the sensitivity of [^18^F] FDG PET/CT and [^18^F] FDG PET/MRI in lymph node metastasis detection in nasopharyngeal carcinoma.

The overall sensitivity of [^18^F]FDG PET/CT for detecting nasopharyngeal carcinoma lymph node metastasis was 0.94 (95% CI: 0.85–0.99). In contrast, [^18^F]FDG PET/MRI exhibited a comprehensive sensitivity of 1.00 (95% CI: 0.94–1.00). The comparison between [^18^F]FDG PET/CT and [^18^F]FDG PET/MRI revealed no significant difference in sensitivity (*p* = 0.20), as illustrated in Figure 7.

### 3.5 Comparing the specificity of [^18^F]FDG PET/CT and [^18^F]FDG PET/MRI for detecting lymph node metastasis in nasopharyngeal carcinoma

**Figure 8:**
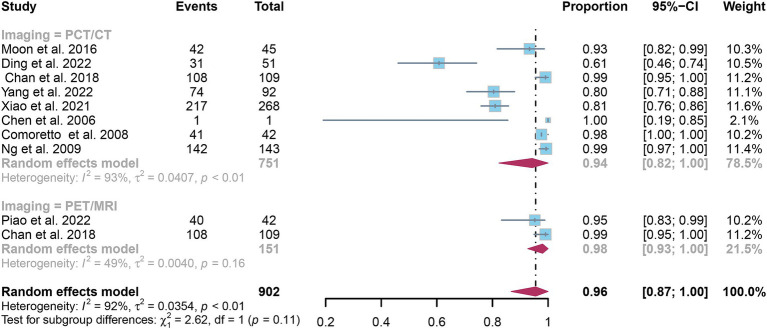
Forest plot comparing the specificity of [^18^F] FDG PET/CT and [^18^F] FDG PET/MRI in lymph node metastasis detection in nasopharyngeal carcinoma.

The pooled specificity for detecting nasopharyngeal carcinoma lymph node metastasis was 0.94 (95% CI: 0.82–1.00) for [^18^F]FDG PET/CT and 0.98 (95% CI: 0.93–1.00) for [^18^F]FDG PET/MRI, as shown in Figure 8. Statistical analysis revealed no significant difference in the overall specificity between [^18^F]FDG PET/CT and [^18^F]FDG PET/MRI (*p* = 0.11).

### 3.6 Publication bias of [^18^F]FDG PET/CT and [^18^F]FDG PET/MRI for detecting lymph node metastasis in nasopharyngeal carcinoma

**Figure 9:**
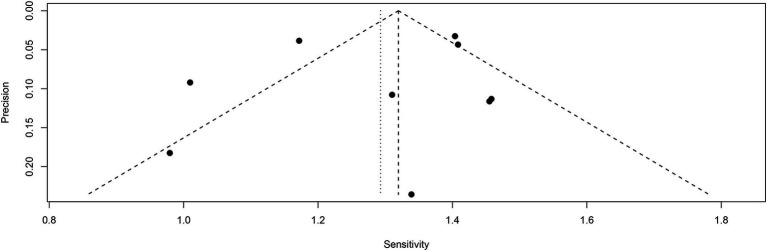
Funnel plot of [^18^F] FDG PET sensitivity for lymph node metastasis in nasopharyngeal carcinoma.

**Figure 10:**
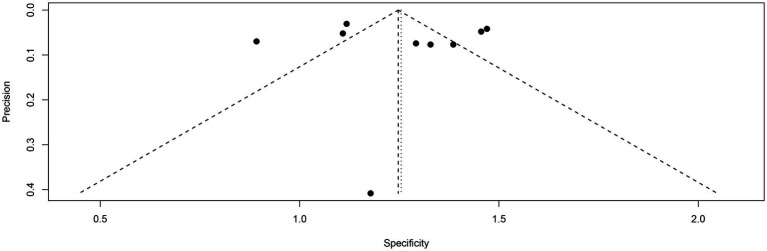
Funnel plot of [^18^F] FDG PET specificity for lymph node metastasis in nasopharyngeal carcinoma.

Funnel plot asymmetry testing indicated no significant publication bias for sensitivity (Egger’s test: *p* = 0.62) or specificity (Egger’s test: *p* = 0.85) in [^18^F]FDG PET (Figures 9, 10).

## 4 Discussion

This meta-analysis significantly advances our understanding by providing a comprehensive comparison of [^18^F]FDG PET/CT and [^18^F]FDG PET/MRI in diagnosing lymph node metastasis in nasopharyngeal carcinoma (NPC), a pivotal factor in effectively managing this condition. Chan et al. (21) posited that PET/MRI might offer superior imaging quality for evaluating lymph node metastasis in NPC. Yet, this claim is met with skepticism by other studies that have demonstrated the substantial effectiveness of [^18^F]FDG PET/CT in similar diagnostic scenarios (27). Such contrasting findings fuel the ongoing debate within the medical community about which modality—PET/CT or PET/MRI—truly represents the best choice for the accurate detection and evaluation of lymph node metastasis in NPC patients. This discourse underscores the necessity for our meta-analysis, aiming to shed light on this debate by methodically comparing the diagnostic accuracies of these two advanced imaging technologies.

Our comprehensive meta-analysis has brought to light the remarkable efficacy of [^18^F]FDG PET in NPC, revealing that both [^18^F]FDG PET/CT and [^18^F]FDG PET/MRI demonstrate equivalently high sensitivity and specificity. This parity in diagnostic accuracy underscores the synergistic potential of the unique imaging capabilities each modality offers. Specifically, PET/CT marries the metabolic detection of PET with the precise anatomical mapping of CT, facilitating an unparalleled accuracy in localizing and characterizing lesions (28). This integration allows for a nuanced visualization of disease activity within its precise anatomical context, thereby enhancing the detection and assessment of NPC. The metabolic imaging aspect of PET highlights areas of increased glucose uptake, indicative of active disease (9), while the CT component provides a detailed anatomical framework, enabling accurate delineation of lesions and their spatial relationships with surrounding structures (29, 30).

Conversely, PET/MRI distinguishes itself with superior soft tissue contrast and functional imaging capabilities (31), offering an enhanced visualization of soft tissue structures that might be critical for the diagnosis and staging of NPC. The high-resolution imaging of MRI, combined with the metabolic insights from PET, presents a comprehensive picture of both the biochemical activity and the structural details of lesions (32). PET/MRI, a dual-modality approach, excels in identifying small or atypically located lymph node metastases, critical for accurate staging and guiding treatment strategies in NPC (12). The complementary strengths of PET/CT and PET/MRI—ranging from detailed anatomical and metabolic insights to exceptional soft tissue delineation—ensure that clinicians are equipped with robust diagnostic tools (33). Ultimately, the strategic selection of the imaging modality, informed by the specific clinical context and the unique diagnostic advantages of each option, is crucial for optimizing the management of nasopharyngeal carcinoma, aiming to improve patient outcomes through precise and informed treatment planning.

A previous meta-analysis by Shen et al. (34) focused on PET or PET/CT in NPC staging, with findings similar to ours regarding sensitivity and specificity. However, they did not compare these results with [^18^F]FDG PET/MRI. Our study fills this gap, offering the first direct comparison between these two modalities.

The considerable heterogeneity was observed in our study (I^2^ = 81% for sensitivity and 92% for specificity). Therefore, we conducted meta-regression and sensitivity analyses to identify the sources of heterogeneity. For specificity, meta-regression indicated that the analytical method (*p* = 0.03) might contribute to heterogeneity. For sensitivity, both meta-regression and sensitivity analyses failed to identify any potential sources of heterogeneity. However, the consistent results post-exclusion of individual studies indicates the robustness of our findings. Other potential factors like patient demographics, disease stage, and technical variations in imaging protocols may also contribute to these heterogeneity (35, 36).

While our results showed similar sensitivity and specificity for both [^18^F]FDG PET/CT and [^18^F]FDG PET/MRI, considerations such as cost, availability, and practicality in clinical settings are crucial. PET/CT, generally more accessible and cost-effective, may be a preferable choice in many situations (37, 38). However, clinical decisions should be individualized, taking into account each patient’s specific clinical situation and the diagnostic capabilities of each modality. In recent years, novel tracers such as FAP-targeting radiopharmaceuticals have emerged as promising tools in oncology, particularly in NPC management. These tracers show high specificity for cancer-associated fibroblasts, which are prevalent in the tumor microenvironment. As highlighted in a recent meta-analysis (39), they hold potential for improving diagnostic accuracy and therapeutic monitoring in NPC. Integrating these novel radiopharmaceuticals into clinical practice could further enhance PET imaging in identifying tumor and lymph node involvement, offering new possibilities for personalized NPC treatment strategies.

Our analysis encounters two primary limitations. Firstly, the inability to identify a definitive source of heterogeneity for the sensitivity of [^18^F]FDG PET, despite meta-regression analysis and sensitivity analysis. Secondly, the limited number of studies included, particularly for PET/MRI, potentially weakens the evidence of our findings. Future research should focus on head-to-head comparisons in larger, diverse cohorts to validate and expand upon our results, enhancing the evidence base for the clinical utility of these imaging modalities in NPC.

## 5 Conclusion

[^18^F]FDG PET demonstrates high sensitivity and specificity in identifying lymph node metastasis in nasopharyngeal carcinoma. Furthermore, [^18^F]FDG PET/CT exhibits comparable sensitivity and specificity to [^18^F]FDG PET/MRI. However, the limited number of studies included in the current analysis may affect the evidence of these findings. Consequently, further research with larger sample sizes and prospective design is essential to corroborate these results.

## Data Availability

The original contributions presented in the study are included in the article/Supplementary material, further inquiries can be directed to the corresponding author.
