# Supplementary material for: [18F]FDG PET/CT versus [18F]FDG PET/MRI in the diagnosis of lymph node metastasis in nasopharyngeal carcinoma: a systematic review and meta-analysis
Source: Front Med (Lausanne). 2024 Oct 16;11:1450526. doi: 10.3389/fmed.2024.1450526 (PMC11521955; doi:10.3389/fmed.2024.1450526)
Supplement: Supplementary file 1 [file Table_1.DOCX]

Supplementary **Table 1** Search strategy in PubMed, Embase, and Web of Science

| Database | Search strategy |
| --- | --- |
| PubMed | ("Positron-Emission Tomography"[Mesh] OR "Positron Emission Tomography" [Title/Abstract] OR "PET"[Title/Abstract]) AND ("Lymphatic Metastasis"[Mesh] OR "Lymph Node Metastasis" [Title/Abstract] OR "LNM"[Title/Abstract] OR " node Metastasis" [Title/Abstract]) AND ( "Nasopharyngeal Carcinoma"[Mesh] OR "Nasopharyngeal" [Title/Abstract] OR "Nasopharyngeal Carcinomas"[Mesh] OR "Nasopharynx"[Title/Abstract]) |
| Embase | ('positron emission tomography'/exp OR ‘Positron Emission Tomography’:ab,ti OR ‘PET’:ab,ti) AND ('lymph node metastasis'/exp OR 'Lymph Node Metastasis':ab,ti OR ' LNM':ab,ti OR 'node Metastasis':ab,ti) AND ( 'nasopharynx carcinoma'/exp OR ' Nasopharyngeal':ab,ti OR 'Nasopharyngeal Carcinomas':ab,ti OR ' Nasopharynx':ab,ti) |
| Web of Science | ((TS=(“positron emission tomography” OR “Positron Emission Tomography” OR “PET”)) AND TS=("Lymphatic Metastasis" OR "Lymph Node Metastasis" OR " LNM" OR “node Metastasis”)) AND TS=("Nasopharyngeal Carcinoma" OR " Nasopharyngeal" OR "Nasopharyngeal Carcinomas" OR "Nasopharynx" ) |
